# Primary care providers’ lived experiences of genetics in practice

**DOI:** 10.1007/s12687-018-0364-6

**Published:** 2018-04-26

**Authors:** Brittany Harding, Colleen Webber, Lucia Ruhland, Nancy Dalgarno, Christine M. Armour, Richard Birtwhistle, Glenn Brown, June C. Carroll, Michael Flavin, Susan Phillips, Jennifer J. MacKenzie

**Affiliations:** 1 Office of Health Sciences Education, Queen’s University, Botterell Hall, Room 217, Kingston, Ontario K7L 2N6 Canada; 2 Queen’s University, 99 University Avenue, Kingston, Ontario K7L 3N6 Canada; 3 Office of Health Sciences Education, Queen’s University, Botterell Hall, Room 217, 18 Stuart Street, Kingston, Ontario K7L 3N6 Canada; 4 Children’s Hospital of Eastern Ontario, 401 Smyth Road, Ottawa, Ontario K1H 8L1 Canada; 5 Centre for Studies in Primary Care, Queen’s University, 220 Bagot Street, P.O.#8888, Kingston, Ontario K7L 5E9 Canada; 6 Department of Family Medicine, 220 Bagot Street, Kingston, Ontario K7L 5E9 Canada; 7 Department of Family & Community Medicine, Mount Sinai Hospital, Granovsky Gluskin Family Medicine Centre, University of Toronto, 60 Murray St., 4th Floor, Box 25, Toronto, Ontario M5T 3L9 Canada; 8 Kingston General Hospital, 76 Stuart Street, Kingston, Ontario K7L 2V7 Canada; 9 Department of Pediatrics, McMaster Children’s Hospital, 1280 Main St. West, 3N11-G, Hamilton, ON L8S 4K1 Canada

**Keywords:** Genetics, Medical, Primary health care, Education, Medical, Genetic services, Rural health services, Education, Continuing

## Abstract

To effectively translate genetic advances into practice, engagement of primary care providers (PCPs) is essential. Using a qualitative, phenomenological methodology, we analyzed key informant interviews and focus groups designed to explore perspectives of urban and rural PCPs. PCPs endorsed a responsibility to integrate genetics into their practices and expected advances in genetic medicine to expand. However, PCPs reported limited knowledge and difficulties accessing resources, experts, and continuing education. Rural practitioners’ additional concerns included cost, distance, and poor patient engagement. PCPs’ perspectives are crucial to develop relevant educational and systems-based interventions to further expand genetic medicine in primary care.

As the gene/environment interaction for common illnesses is being unraveled, translating the “new genetics” into clinical practice is often absent or delayed, resulting in preventable morbidity and mortality from common conditions (Wilde and Behr 2013; Couch et al. 2017) such as inherited cancers, diabetes, and cardiac disorders. Primary care providers (PCPs) are ideally situated to support families by facilitating genetic risk assessment, responding to patient concerns, and providing management (Feder and Modell 1998; Watson et al. 1999; Metcalfe et al. 2002; Carroll et al. 2003; Qureshi et al. 2004; Blashki 2005; Telner et al. 2008; Mathers et al. 2010; Liaw 2010; Houwink et al. 2011; Petrou 2013), and it is in their offices that most patients will first raise genetic issues. Thus, PCPs need to be equipped to integrate genetics into their practices (Watson et al. 1999; Burke and Emery 2002; Qureshi et al. 2004; Khoury et al. 2007; Telner et al. 2008; Houwink et al. 2011).

PCPs’ views about the clinical utility of genetics continue to evolve. Prior research exploring PCPs’ perspectives of genetics in international settings has suggested (Feder and Modell 1998; Emery et al. 1999; Fry et al. 1999; Watson et al. 1999; Fetters et al. 1999; Williamson and Robertson 1999; Greendale and Pyeritz 2001; Metcalfe et al. 2002; Carroll et al. 2003; Qureshi et al. 2004; Bottorff et al. 2005a, b; Guttmacher et al. 2007; Telner et al. 2008; Mathers et al. 2010; Liaw 2010; Weir et al. 2010; Houwink et al. 2011) that PCPs perceive themselves to be “gatekeepers” to genetics by assessing risk (Emery et al. 1999; Fry et al. 1999; Williamson and Robertson 1999; Carroll et al. 2003; Bottorff et al. 2005a, b; Liaw 2010), referring patients, and facilitating management plans (Feder and Modell 1998; Fry et al. 1999; Watson et al. 1999; Fetters et al. 1999; Carroll et al. 2003; Telner et al. 2008). Perceived barriers included gaps in provider knowledge and skills (Emery et al. 1999; Fry et al. 1999; Watson et al. 1999; Greendale and Pyeritz 2001; Metcalfe et al. 2002; Carroll et al. 2003; Qureshi et al. 2004; Bottorff et al. 2005a, b; Guttmacher et al. 2007; Mathers et al. 2010; Liaw 2010; Weir et al. 2010; Houwink et al. 2011), time, cost, and inadequate management guidelines (Emery et al. 1999; Metcalfe et al. 2002; Carroll et al. 2003; Weir et al. 2010; Houwink et al. 2011). PCPs endorsed practical education (Liaw 2010) about the diagnosis and management of common genetic disorders (Fetters et al. 1999; Greendale and Pyeritz 2001; Metcalfe et al. 2002; Telner et al. 2008; Houwink et al. 2011) through undergraduate, postgraduate (Metcalfe et al. 2002; Guttmacher et al. 2007), and continuing education (CE) (Fetters et al. 1999; Metcalfe et al. 2002; Bottorff et al. 2005a; Guttmacher et al. 2007). Perceived needs have included accessible, reputable sources of information (Emery et al. 1999; Metcalfe et al. 2002; Carroll et al. 2003; Qureshi et al. 2004; Bottorff et al. 2005a, b), referral and management guidelines, and accessible support from genetic specialists (Fry et al. 1999; Qureshi et al. 2004; Hamilton et al. 2014; Carroll et al. 2016). Consensus suggests that (Guttmacher et al. 2007), although genetics should be included in PCP patient care repertoire, there has been no systemic approach such as support for enhanced electronic medical records, and CE. Although attempts have been made to address these concerns, the integration of genetics into primary care practice remains a challenge (Carroll et al. 2016).

While the needs and barriers related to the implementation of genetics in primary care have been explored, limited information about rural settings exists. Two small studies in rural USA and Wales identified practical difficulties with referral to genetic specialists including distance, access to transportation, and cost, as well as a lack of PCP and/or patient awareness and interest in genetics (Koil et al. 2003; Iredale et al. 2005). No studies have specifically examined the perspectives of rural Canadian PCPs regarding their role in genetic medicine, barriers to integrating genetics into rural practice, and specific educational needs.

The purpose of this study was to explore genetics in primary care from the perspective of both rural and urban PCPs. The findings are intended to provide a foundation to develop innovative strategies to optimize PCPs’ ability to provide genetic care.

## Methods

A qualitative phenomenological methodology (Creswell 2013) was used to describe and analyze the lived experiences of PCPs with respect to the following: (1) how they perceive their role in genetic medicine, (2) facilitators and barriers to integrating genetics into practice, and (3) the future relevance of genetics in primary care. Ten key informant interviews and three focus groups (FGs) were conducted. Clearance was received from a Canadian University/Teaching Hospital Health Sciences Research Ethics Board.

### Setting and population

#### Interviews

Using stratified purposeful sampling, participants were recruited to represent a cross section from rural and urban settings in Southeastern Ontario (SEO) based on advice from the research committee. Rural was defined as a population density under 400 people/km^2^ (Statistics Canada 2009). Key informants (half rural) included one health care administrator, one clinical geneticist, one nurse practitioner, one public health administrator, two genetic counselors, and four PCPs with overlapping clinical, educational, and administrative roles.

#### Focus Groups

Invitations for FG were sent to urban and rural Family Health Teams in SEO. Once interest was established, a health administrator from that Family Health Team invited all PCPs in their region. Participants were PCPs practicing in SEO and represented rural (FG2 and FG3; *n* = 14) and urban settings (FG1; *n* = 5). FG participants included females and males between 30 and 60 years old with a minimum of 5 years in practice.

### Data collection

A research assistant (RA) conducted semi-structured interviews (*n* = 10) over 5 months (March to July 2012) to inform the design of the FG script. The protocol included open-ended questions with probes and was based on a literature review and suggestions from the research team.

The RA and participants reviewed the transcripts. Four out of ten participants verified their transcripts. The remainder did not respond. No data were redacted. Interview results are reported as the initial FG script.

Three FGs were conducted over 5 months (January to June 2013). FG scripts were semi-structured and included open-ended questions with probes. The scripts were modified as new data emerged. A RA conducted the FGs, while the principal investigator (PI) recorded field notes.

FGs and interviews were audio-recorded and transcribed verbatim. Identifying data were removed prior to analyses. Data were stored and managed using NVivo10.

### Data analysis

Thematic analysis was used to develop a deep understanding about participants’ perceptions and experiences. Analysis was iterative and protocols were revised throughout the data collection process. Three RAs independently coded the interviews and FGs. Codes were compared and revised to ensure inter-rater dependability. The final codebook resulted in 78 codes organized into 14 categories and five themes. In the case of inconsistencies, the PI analyzed the scripts and inconsistencies were discussed with the RAs until consensus was reached. The findings were deliberated with the entire research team.

Member checking of the interviews, analysis by multiple RAs, input of the research team, distanced RAs, and correlation with the literature ensured the trustworthiness and consistency of data.

## Results

Fourteen categories and five underlying themes emerged from the FG (Table 1). Tables 2, 3, 4, 5, and 6 provide selected quotes that support each theme.

**Table 1:** Summary of themes and categories

Themes	Categories
Role of genetics in primary care	Encounters with genetics in practice
	Genetics impact on treatment
	Value of genetic care
PCP responsibilities	Genetic care through family history
	Referral to genetic testing
	Patient care
Barriers	System-related
	Provider-related
	Patient-related
Needs	Education
	Resources
	Support
Future of genetic care	Greater role and demand for genetic care
	Future applications of genetics care in practice

**Table 2:** Role of genetics in practice

Category	Selected quotes
Encounters with genetics in practice	“The only time it comes up is if someone’s planning a pregnancy… or if suddenly a whole bunch of family members have cancer. Those are the only instances where I’ve had anybody raise any kind of genetic questions…. or if a baby’s been found to have some kind of abnormality than that kind of opens it up.” (FG1)
Genetics impact on treatment	“Genetics is becoming part of everything in family medicine. It’s just permeating everything you do…. It’s going to be tricky for us to keep track.” (FG3)
Value of genetic care	“We need to know what conditions are actually cost effective to screen for, because if we can prevent or manage then outcomes are better…. You have to look at the big picture… is the test expensive?” (FG1)

**Table 3:** Primary care provider responsibilities in genetics care

Category	Selected quotes
Genetic care through family history	“Patients with diseases that run in families… Figuring if they’re a baseline risk or if they’re an elevated risk… how to work that up is probably what I do most.” (FG3)
Referral to genetic testing/counseling	“Like so many areas of medicine, the role of the family physician is to help decide whether their concerns are legitimate or not. Make the appropriate referral if it is [or] try and reassure them that it is a misplaced concern.” (FG2)
Patient care	“People oftentimes don’t understand the implications of [genetics]…. You have to sit down and talk to them…. I want them to understand that there are false positives and false negatives… sometimes you have to spend more time with …people who aren’t familiar…. They haven’t talked about it, or they don’t really understand it and so it takes quite a bit of time.” (FG1)

**Table 4:** Barriers to genetics care in practice

Category	Selected quotes
System-related	“Patient that had a family history of... a genetic abnormality leading to a clotting disorder. I sent them out for the test and … it would have been $300 to the patient….A lot of [conditions], even if they come up, aren’t relevant to us as primary care physicians because they just aren’t on the public funds.[Patients] have to be referred to the hospital to be tested.” (FG1) “We talked about [a resource] years ago, with the family health team, but the funding just fell apart. But [if] you had somebody that [could] check out [information sites on the web], make sure it’s valid information, [keep us] up to date….” (FG2) “I have come across patients that have been reluctant to travel … for a consult.” (FG2) “There’s this phenomenon in smaller communities where, when certain services aren’t available for a long period of time, you learn to get along without them…. The availability of the service doesn’t necessarily mean more people will get better….” (FG2)
Provider-related	“I haven’t had anybody say, ‘no I don’t want to go’. I’ve had tons [of patients] come back and say to me ‘so now what should I do.’ They ask me what to do… not [the geneticist]. Which puts you in a spot…. the biggest barrier for me would be not having the knowledge on enough of the appropriate diseases.” (FG3) “It does take quite a bit of time … a lot of time counselling patients…and if you were to think about how many other syndromes and conditions… might arise to start counseling…not to say that we shouldn’t be doing it…” (FG1) “I think things change quickly and we’re not always aware of… which genetic tests… we can do…I would not know what to order… I don’t think we have a good enough understanding, at least I don’t, of genetics.” (FG2)
Patient-related	“A lot of patients I saw sometimes are a bit apprehensive. And maybe with good reason… if they’re thinking, ‘what are the implications of finding this information out’, [or]if they don’t have insurances in place…. Sometimes when I talk to patients about [testing] they’re hesitant. Sometimes family members don’t want to cooperate by participating… There’s a whole unknown for the patient…sometimes they [don’t want] to jump on board with finding out more.” (FG1)

**Table 5:** Needs to improve genetics in primary care practice

Category	Selected quotes
Education	“If all the information about the expansion of genetic knowledge and testing capabilities is true, it sounds to me like it’s something I’d like to know about. There are lots of things that came up during my career that I didn’t learn about in medical school and yet it’s important to try and figure out what’s going on and be at least on the curve if not ahead of the curve.” (FG1)
Resources	“I’m still not there… but you see the studies coming out, you keep it in the background and then eventually lose track. It would be nice to have a regular update of what’s in the pipeline and where it is and what’s the evidence behind [it].” (FG3) “[A screening] questionnaire [could] be mailed out to the patient and they [could]do it virtually or online…. We as physicians could access a website that would allow us [to send surveys] for various conditions, or patients themselves could [access surveys], or be mailed [surveys] if they preferred that.” (FG2)
Support	“Prevention is concentrated on, is very mandated and very specifically pushed very heavily by the Ministry….If it doesn’t fall into the focus of the Ministry, it’s essentially ignored…. I’ll admit [that] I’m far more knowledgeable about things that are on the agenda than things that are not.” (FG1)

**Table 6:** Future of genetic medicine in primary care

Category	Selected quotes
Greater role of genetics in practice and increased demand for genetic care	“I think technology will march on and there’ll be a lot more screening tests available. I hope [they] will have a lot more direction attached to them…we will certainly hear more as time passes. “(FG2)
Future applications of genetics care in practice	“There are a lot more things to do now then there were two years ago and somehow we managed to do that…. more work in teams, we use allied health professionals … share the load on a number of things…. one way of coping with the increased number of things there are to do…. giving up some stuff too – aren’t we? There are some things that we used to do that [we know] aren’t useful… we lose some things as we gain more knowledge.” (FG1)

### Role of genetics in practice

PCPs reported limited encounters with genetics in their practices, but had not explicitly identified some activities such as family history assessment as practicing genetic medicine (Table 2). Most clinical exposures described by PCPs related to pregnancy or hereditary cancers; rare genetic conditions were less commonly encountered. PCPs were aware that genetics permeates many health conditions and used genetic information as a screening tool. Although knowledge about the genetic basis of a condition was considered beneficial, considerations about the interpretation of genetic test results, clinical utility, cost-effectiveness, and communication strategies were areas that PCPs felt needed additional clarification. Rural and urban PCPs had similar perspectives about the value of genetics in primary care.

### Primary care provider responsibilities

PCPs endorsed a responsibility to take family histories, assess risk, determine appropriate management strategies, and facilitate referrals (Table 3). PCPs viewed their role to include responding to patient concerns and educating and counseling patients about ethical, psychological, and medical aspects of genetics.

Regarding access to genetic services, some PCPs ordered genetic testing for common conditions such as hemoglobinopathies. PCP comfort with genetic testing varied depending on personal training and experience. However, concern was raised that further genetic responsibilities would be downloaded to PCPs without sufficient support. One participant believed that genetic medicine was outside of a PCP’s scope and the responsibility of a geneticist or a non-genetics specialist who could refer on to genetics. Uncertainty about responsibility and the notion of specialist to specialist referral is important to acknowledge as it is counter to traditional views of practice, and may not be a consistent expectation so could result in gaps in care.

### Barriers to genetic care

System-, patient-, and provider-related barriers to integrating genetics into patient care were described (Table 4).

Systemic barriers included access to resources such as trained staff and up-to-date materials, costs to patients for community-based genetic testing, and access to timely communication from referrals about follow-up plans especially for rural PCPs.

Perceived barriers to patient uptake of genetic counseling and testing included apprehensiveness, lack of understanding of genetics, limitations of genetic testing, implications of results, impact on insurance, and limited awareness about management options. Time, transportation, finances, and missed employment were deterrents, primarily for rural patients.

PCPs’ lack of confidence in their genetic knowledge may have resulted in missed opportunities for genetic care. Time to offer effective genetic care to patients, a perceived need to justify genetic testing, and costs for community-based genetic testing were considered problematic. These concerns influenced PCPs’ and patients’ decision-making about whether a genetics referral was worthwhile. Although telemedicine was useful, rural PCPs emphasized the value of face-to-face counseling. Due to delays in instituting new services, PCPs reported that rural communities have learned to manage “without.” Thus, when expanded services become available, the service may not be utilized optimally for some time.

### Needs to improve genetics in primary care practice

PCPs identified a need for education, resources, and supports to aid them in improving the genetics care they could offer (Table 5).

Foundational genetic education in undergraduate and postgraduate medical curricula was described as limited, resulting in PCPs’ feeling unprepared for genetic aspects of health care.

PCPs’ primary interests included information about current resources, tests, and referral guidelines. Participants emphasized a need for an array of CE options including email updates, pamphlets, mail-outs, and an online database of genetic conditions. Urban PCPs preferred in-person CE sessions. Timely access to an expert by telephone or email was suggested in rural settings due to limited opportunities for face-to-face sessions and informal interactions with colleagues. Industry sponsorship to attend CE was accessible, primarily for rural PCPs, but at risk for bias.

An electronic medical record (EMR) with alerts for genetic risks, online family history tools, and telemedicine were suggested and access by both PCPs and patients endorsed.

PCPs emphasized that improved health care is best adopted when supported by medical administration and/or a governing body. A central body to co-ordinate ongoing guidelines and CE for genetics, and appropriate reimbursement for genetic care was suggested. PCPs concurred that current health care funding models were insufficient to sustain genetics in primary care and suggested support could be elicited from local medical schools and hospitals.

### Future of genetic medicine in primary care

Although PCPs discussed challenges, public awareness and advances in genetic medicine are increasingly driving genetics into daily practice (Table 6). Practice patterns were described as shifting such that new advances would replace prior standards. The social implications of genetic testing especially direct to consumer testing and celebrity endorsement raised concerns, but it was anticipated that management of these issues would be part of future practice. Urban and rural PCPs expected genetic advances to impact their provision of diagnostic testing, screening, surveillance, treatment, and personalized medicine.

### Process results

The FGs prompted discussions about personal experiences with integrating genetics into practice. Subsequently, PCPs worked with the regional genetic center to try to improve local support. Although an unintended outcome, ongoing relationships affirm the importance of communication and collaboration among health care professionals.

## Interpretation

PCPs describe increased interest in genetics and expect their responsibilities for education, counseling, testing, and referrals to specialists to continue to evolve as clinical utility expands. In spite of increased PCP interest and perceived level of responsibility (Mikat-Stevens et al. 2014), both rural and urban PCPs continue to report inadequate preparation and support for their roles. Although workload issues need attention, PCPs endorsed the idea that new standards would replace the status quo. PCPs expect their responsibilities for education, counseling, testing, and referrals to specialists to evolve as the clinical utility of genetics expands. Although workload issues need attention, PCPs also thought that prior activities would be replaced with new standards. A lack of consensus about responsibilities for providing genetic care combined with the barriers described, especially for rural PCPs, challenged those attempting to embed genetics into their practice. PCPs’ perspectives and comfort levels may be influenced by varied exposure to genetic medicine in training and practice consistent with the findings of Julian-Reynier et al. (2008). Rural practitioners faced additional barriers related to patients’ ability to travel to specialized centers, access to CE, formal and informal support from colleagues with genetic expertise, and provider/patient interest in genetic contributions to health. However, PCPs acknowledge that rural concerns are not unique to genetics. Thus, our findings align with research in other medical systems and contribute new insights into the perspectives of rural Canadian PCPs (Feder and Modell 1998; Emery et al. 1999; Fry et al. 1999; Watson et al. 1999; Fetters et al. 1999; Williamson and Robertson 1999; Greendale and Pyeritz 2001; Metcalfe et al. 2002; Carroll et al. 2003; Qureshi et al. 2004; Bottorff et al. 2005a, b; Guttmacher et al. 2007; Telner et al. 2008; Julian-Reynier et al. 2008; Mathers et al. 2010; Liaw 2010; Weir et al. 2010; Houwink et al. 2011, 2012; Hamilton et al. 2014; Mikat-Stevens et al. 2014; Delikurt et al. 2015; Carroll et al. 2016)(Feder and Modell 1998; Emery et al. 1999; Fry et al. 1999; Watson et al. 1999; Fetters et al. 1999; Williamson and Robertson 1999; Greendale and Pyeritz 2001; Metcalfe et al. 2002; Carroll et al. 2003, 2016; Qureshi et al. 2004; Bottorff et al. 2005a, b; Guttmacher et al. 2007; Telner et al. 2008;; Mathers et al. 2010; Liaw 2010; Weir et al. 2010; Houwink et al. 2011, 2012; Hamilton et al. 2014; Mikat-Stevens et al. 2014; Delikurt et al. 2015).

To ensure PCPs have the skills, strategies, and supports to adequately care for their patients, an explicit understanding of the successes and barriers to the integration of genetics into primary care is critical. Issues to consider are multifaceted and include effective medical education and continuing education (CE), adequate infrastructure, allocation of roles and responsibilities, engagement of health administrators, and availability of just-in-time, up-to-date resources. Rural PCPs may provide a broader scope of care than their urban counterparts, but report less access to CE, follow-up communications from specialists, and informal consultation with colleagues. The narrative of a culture of “making do without” in rural communities compounds the challenge of introducing new services because a shift in practice patterns and public expectation is needed before new services are utilized as intended and become the new standard of care. Factors compounding the challenges faced by PCPs include advances in genetic medicine that have the potential to outpace the ability of PCPs to keep up to date. These advances include the rapid expansion of and public interest in for–profit genetic testing such as direct-to-consumer tests (LifeLabs Medical Laboratories 2017), local clinics offering user-pay genetic testing, and genetic profiles to direct drug therapy. Concerns with patient awareness, interest, compliance, and apprehension may limit physician and patient engagement (Delikurt et al. 2015). Barriers to the provision of genetic care have the potential to result in incorrect provision of genetic counseling, sub-optimal use of genetic testing, and missed referrals, resulting an increase in morbidity and mortality. Cases traditionally referred to genetics are now being seen and managed by PCPs or are expected to be, at least by some (Houwink et al. 2011; Reed et al. 2015). Therefore, it is incumbent on medical educators and regulators to ensure PCPs have the appropriate skills and resources to provide care for their patients.

Early integration and ongoing exposure to genetics in medical training have the potential to support future genetics care. Although medical educators recognize that medical school genetics curricula must transition from a focus on basic science to clinical care (Challen et al. 2005; Schmidtke et al. 2006; Burke and Kirk 2006), medical education trails behind scientific and therapeutic advances. In spite of the literature, PCPs continue to receive little if no training in clinical genetics, and medical curricula do not address the practicalities of integrating genetics into practice (Bottorff et al. 2005a; Harris et al. 2006; Houwink et al. 2011, 2013). Although the accredited objectives established by the Medical Council of Canada for undergraduate medical education include basic genetic skills (Medical Council of Canada 2017), the degree to which these are integrated into medical school curricula varies (personal experience). Genetics education continues to compete with other topics, so a targeted approach would include the incorporation of genetics into case-based learning and clinical skills among other areas. At the postgraduate level, genetics integration into practice varies depending on location and practice type although genetics is endorsed as important in residency training programs. Thus, postgraduate genetics education may depend on the extent to which genetics is practiced by PCP supervisors/mentors as well as the type of patient encounters that the resident is exposed to (Telner et al. 2008, 2017).

PCPs report interest in genetics CE and some programs have improved PCP confidence but publications are limited to small samples of volunteers (Carroll et al. 2011, 2016; Paneque et al. 2015). Although CE has demonstrated some success, the extent to which practice has changed is difficult to objectively measure and the optimal methods remain part of the current discourse. Skills considered important to effectively impact patient care include increased awareness of genetic services, up-to-date knowledge about relevant genetic issues, a shift in perspective about the utility of genetic testing, and increased confidence (Carroll et al. 2009). Formal genetic education and CE have demonstrated an increase in knowledge integration, confidence, and behaviors related to genetic practice skills when blended learning courses and online modules were used and attention was paid to the practicalities of integration of genetics into primary care practice. These studies suggest that educational interventions should be patient-centered, case-based, transferable to practice, and easily accessible (Houwink et al. 2013; David et al. 2015; Reed et al. 2015; Carroll et al. 2016). Thus, websites, online applications, and an electronic medical record that includes genetics have the potential to improve genetics skills within daily practice settings. Further strategies could include providing patient information letters about rare conditions to share with PCPs, PCP involvement in in-person or virtual (telehealth) appointments with their patients and in multidisciplinary clinics. In the author’s experience, this has improved the ability of non-genetics physicians to provide genetic care and to develop a practical working relationship with their local genetics program. Regardless of the educational interventions provided, it is imperative that PCP’s genetic knowledge and skills development are thoughtfully aligned with the core genetic competencies and CE learning requirements within their profession and clinical settings (Houwink et al., 2011).

From a health systems perspective, many opportunities exist including the addition of genetic counselors to family health teams, rotating consultant geneticists to attend PCP practices, shadowing PCPs who already integrate genetics into practice, and genetics professionals attending case rounds in PCP practices to discuss potential genetic aspects of cases presented. Other models of care to target rural barriers include the addition of genetics nurses/counselors into regional units such as public health units serving rural regions, a model that has had some success in Ontario (Health Sciences North 2013). Although PCPs report a paucity of guidelines, only 16% report using them (Vig et al. 2009). Therefore, the benefit of additional guidelines is questionable unless these are linked to specific billing codes or standards of care. All of these strategies require the engagement of health administration to provide the infrastructure and resources needed.

Those PCPs attempting to embed genetics into their practices describe a lack of consensus about roles and responsibilities for genetic care combined with a varied comfort level with genetic knowledge and management. PCPs themselves had varied perspectives likely related to their exposure to genetic care in their training and current practice environment. This lack of consensus about the role of the PCP for genetic care suggests that the health system needs to further define genetic expectations for community practitioners and to provide the required infrastructure, including training and resources, to provide that care. While expectations may vary by region and practice type, alignment with sub-specialists is necessary within systems to ensure that patient needs are addressed in an organized manner. With 75 clinical geneticists in Canada (Canadian Medical Association [Internet]) and about 200 genetic counselors in Ontario who need to focus, at least in part, on rare diseases and emerging therapies (Shuman, 2017, written communications), much, if not most, of genetic care will fall to other health care providers.

Overall, the relationship of medical education/CE to the integration of genetics into practice may not be as linear as providing additional courses and media campaigns but may be related to an implicit understanding about the impact of genetics on human health and considerations about how to situate genetics into practice (Robins and Metcalfe 2004). Thus, a repositioning of genetics in the culture of patient care needs to occur—one that includes an examination of roles and responsibilities of PCPs, explicit expectations of health care providers and patients, engagement of health administration, and an authentic recognition of the benefits and limitations of genetics in medicine.

### Conclusion and future directions

PCPs, both urban and rural, endorse the importance of genetics in primary care. The current crisis is the provision of timely and efficient genetic care. In order for this to be provided, PCPs must have the ability to assess genetic risk, provide a consistent level of genetic care including surveillance and testing, and identify and refer those cases beyond their scope of practice. Although interest in genetics has increased over the last several years due to expanded clinical utility, accessibility of testing, and public interest, the challenges PCPs report remain consistent with prior research in spite of some efforts to expand genetics CE. We hope to provide foundational knowledge to support innovative approaches to facilitate the integration of genetics into primary care practice including but not limited to the design and evaluation of CE and resources that address cultural as well as systemic issues. CE initiatives could include “just-in-time” educational resources, an expert hotline, and the integration of genetic counselors/visiting geneticists into primary health care teams. Critical to the development of strategies to integrate genetics into primary care is an improved understanding of the value the public and our health systems place on genetic health and aligning these expectations with those of PCPs.
